# Bilateral Brucellar Sacroiliitis Mimicking Tuberculous or Inflammatory Spondyloarthropathy: A Case Report

**DOI:** 10.1002/ccr3.72981

**Published:** 2026-06-18

**Authors:** Zia Ullah, Zain Ul Abedeen, Muhammad Kashif Habib, Naeem Ullah, Abad Ur Rehman

**Affiliations:** ^1^ Department of Medicine Saidu Medical College Swat Pakistan; ^2^ Department of Medicine Spinghar University (Kabul Campus) Kabul Afghanistan

**Keywords:** brucellosis, case report, magnetic resonance imaging, osteoarticular brucellosis, sacroiliitis

## Abstract

Brucellosis is a zoonotic infection, endemic in rural areas. It is caused by Brucella species, which are transmitted through the intake of unpasteurized dairy products and contact with infected animals. Sacroiliac joint involvement is common in brucellosis and typically presents as unilateral sacroiliitis. Contrarily, bilateral sacroiliitis is less typical and may mimic tuberculosis, pyogenic infection, or inflammatory spondyloarthropathy. We report a case of a 19‐year‐old man who presented with a one‐month history of bilateral buttock pain radiating to the legs and a 2‐week history of fever, chills, rigors, and febrile diaphoresis. He reported unpasteurized milk intake. Physical examination revealed bilateral sacroiliac joint tenderness, and Patrick's and Gaenslen's provocation tests elicited pain in both sacroiliac joints. Laboratory findings showed leukocytosis, elevated CRP, and ESR. Standard agglutination testing was positive for anti‐Brucella antibodies, and MRI confirmed bilateral sacroiliitis, along with erosive changes and marrow edema. A diagnosis of brucellosis complicated by bilateral sacroiliitis was proposed. The patient was started on doxycycline, co‐trimoxazole, and gentamicin. Gentamicin was used for 22 days, while doxycycline and co‐trimoxazole were continued for a total planned duration of 13 weeks. During follow‐up visits (day 15 and day 45), the patient demonstrated clinical improvement, evident by laboratory parameters, pain improvement, no fever, difficulty walking, and medication‐related adverse effects. This case emphasizes that clinicians in endemic areas must consider brucellosis as a differential diagnosis in patients with bilateral lower back or buttock pain. MRI findings and serology testing are useful in early recognition. While blood cultures, PCR, and biopsy are necessary for definitive diagnosis.

## Key Clinical Message

Clinicians in endemic regions should consider brucellosis in patients with bilateral sacroiliitis, fever, and raw dairy exposure history. Bilateral involvement is less typical and may mimic tuberculous and autoimmune spondyloarthropathies on presentation, posing a significant diagnostic challenge.

**Abbreviations**
CRP: C‐reactive protein
ESR: Erythrocyte sedimentation rate
L4–L5: Lumbar intervertebral level L4–L5
L5–S1: Lumbar–sacral intervertebral level L5–S1
MRI: Magnetic resonance imaging
PCR: Polymerase chain reaction
SAT: Standard Agglutination Test
WBC: White blood cell
ZN: Ziehl–Neelsen

## 1 Introduction

Brucellosis, also called Malta fever, is a zoonotic infection caused by facultative, gram‐negative coccobacilli of the genus Brucella. Its transmission occurs through ingestion of unpasteurized milk and its products or direct contact with an infected animal, which is why it is more prevalent in rural areas where animal husbandry and agriculture are common practices [1, 2]. Brucellosis exhibits a variable clinical presentation, ranging from fever (the most common symptom), decreased appetite, and fatigue to some organ‐specific complications, such as arthritis, pancytopenia, jaundice [1], hepatosplenomegaly, genitourinary, and respiratory problems [3, 4].

Sacroiliitis is the inflammation of the sacroiliac joint. It can arise from several causes, including autoimmune, trauma, infections, and gout [5]. Osteoarticular involvement is a recognized focal complication of brucellosis, encompassing arthritis, spondylitis, and sacroiliitis [6]. Brucellar sacroiliitis typically presents with unilateral pain in the lower back or hip region, sometimes radiating to the legs (unilateral sacroiliitis). It often leads to joint damage and disability. In contrast, bilateral sacroiliac involvement in brucellosis is less typical and may pose diagnostic challenges [1, 7]. It can mimic tuberculous sacroiliitis, pyogenic infection, or inflammatory spondyloarthropathy, necessitating a thorough clinical evaluation [7, 8]. For management, it requires a combination antibiotic therapy, and treatment often yields a good prognosis, especially when diagnosed early and treated adequately [6].

Bilateral sacroiliac joint involvement in brucellosis, as in our case, is an uncommon and diagnostically misleading presentation, making such cases noteworthy. Our case report highlights the need for detailed exposure history, targeted serological testing, careful interpretation of magnetic resonance imaging, and exclusion of both tuberculosis and inflammatory spondyloarthropathy.

## 2 Case Report

### 2.1 Patient History

We present a case of a 19‐year‐old male patient who presented with a one‐month history of bilateral buttock pain along with fever for the last 2 weeks (day 1). The patient reported pain as dull, continuous, radiating to the legs, aggravating with movements, coughing, and sneezing, but relieved by rest and did not necessitate any medication intake. The pain severity was 6/10 and caused mild difficulty in walking and prolonged sitting. He developed fever 2 weeks back, which was gradual in onset and low grade initially, but soon became high grade with associated profuse sweating, rigors, and chills. The patient started taking paracetamol for fever, which also partially relieved his pain. He was a resident of a rural area with no recent history of travel and animal contacts apart from consuming unpasteurized cow milk. He denied any urinary symptoms, sexual symptoms, weight loss, night sweats, or any prior medical or family history of zoonotic infections. He had not received antibiotics before the presentation.

### 2.2 Physical Examination

On examination, he was febrile but hemodynamically stable, with no respiratory distress. His pulse, blood pressure, and oxygen saturation were normal. The patient walked in mild discomfort, but his overall gait was normal. The buttock region did not show any associated skin changes, including redness, warmth, swelling, discharge, or discoloration. The hip joint had a normal range of motion, and the relevant physical examination did not reveal any positive findings. Direct sacroiliac compression, Patrick's, and Gaenslen's provocation tests reproduced pain over both sacroiliac joints. These findings raised suspicion of sacroiliac‐joint involvement and led to further evaluation with laboratory investigations and MRI for diagnostic confirmation. Additionally, lumbar spine movements were also restricted. The sensory, motor, and vascular examinations of the lower limbs were not significant. No lymphadenopathy, hepatosplenomegaly, or other findings were noted on systemic examination. Based on history and physical examination, our differential diagnosis included the following: pyogenic sacroiliitis, spondyloarthropathy, traumatic sacroiliitis, tuberculous sacroiliitis, disc pathology, brucellar sacroiliitis, and mechanical lower back pain.

### 2.3 Investigations and Diagnosis

Laboratory investigations indicated mildly raised WBCs [11.7 × 10^9^/L (reference range: 4.0–11.0 × 10^9^/L)] and inflammatory markers—including C‐reactive protein [6.6 mg/dL (reference range: < 1.0 mg/dL)] and ESR [25 mm/h (reference range: < 15 mm/h)]—and normal renal function tests and serum electrolytes. Standard agglutination testing was positive for anti‐Brucella antibodies, with antibody titers against *Brucella abortus* antigen of 1:320 and *Brucella melitensis* antigen of 1:160 (Table 1). MRI of the lumbar spine was advised, which showed erosive changes and marrow edema in the bilateral sacroiliac joints, more marked on the left side (Figure 1). Based on the history, clinical features, positive Brucella serology, raised inflammatory markers, and MRI findings, a diagnosis of brucellosis complicated by bilateral sacroiliitis was proposed. Tuberculous sacroiliitis was deemed unlikely given the absence of weight loss and night sweats, as well as the MRI was not showing any abscess formation or destructive changes which are typical of advanced spinal tuberculosis. Pyogenic sacroiliitis was considered less likely because there was no overlying skin infection, discharge, marked systemic toxicity, or rapid clinical progression. Inflammatory spondyloarthropathy was less likely because there was no history of psoriasis, uveitis, inflammatory bowel disease, enthesitis, preceding genitourinary symptoms, or neurologic compromise. Blood culture, PCR, and image‐guided biopsy, which were necessary for microbial confirmation and definitive diagnosis, were not performed due to financial constraints and/or lack of availability. This has been explicitly acknowledged in the limitation section. Nevertheless, history, serology, and MRI reports supported early diagnosis and helped guide treatment decisions.

**TABLE 1:** Laboratory trends at presentation (day 1), day 15, and day 45.

Test	Day 1	Day 15	Day 45	Reference value
WBC count	11.7 × 10^9^/L	9.3 × 10^9^/L	7.3 × 10^9^/L	4.0–11.0 × 10^9^/L
Hemoglobin	138 g/L	136 g/L	139 g/L	130–170 g/L
Platelet count	285 × 10^9^/L	276 × 10^9^/L	260 × 10^9^/L	150–450 × 10^9^/L
CRP	6.6 mg/dL	1.1 mg/dL	0.4 mg/dL	< 1.0 mg/dL
ESR	25 mm/h	16 mm/h	10 mm/h	< 15 mm/h
Serum creatinine	79.6 μmol/L	84 μmol/L	77.8 μmol/L	62–1115 μmol/L
BUN	5 mmol/L	5.7 mmol/L	4.6 mmol/L	2.5–7.1 mg/dL
SAT against *Brucella abortus* antigen	1:320 titer	—	—	Negative or < 1:160 titer
SAT against *Brucella melitensis* antigen	1:160 titer	—	—	Negative or < 1:160 titer

Abbreviations: BUN, blood urea nitrogen; CRP, C‐reactive protein; ESR, erythrocyte sedimentation rate; SAT, standard agglutination test; WBC, white blood cell.

**FIGURE 1:**
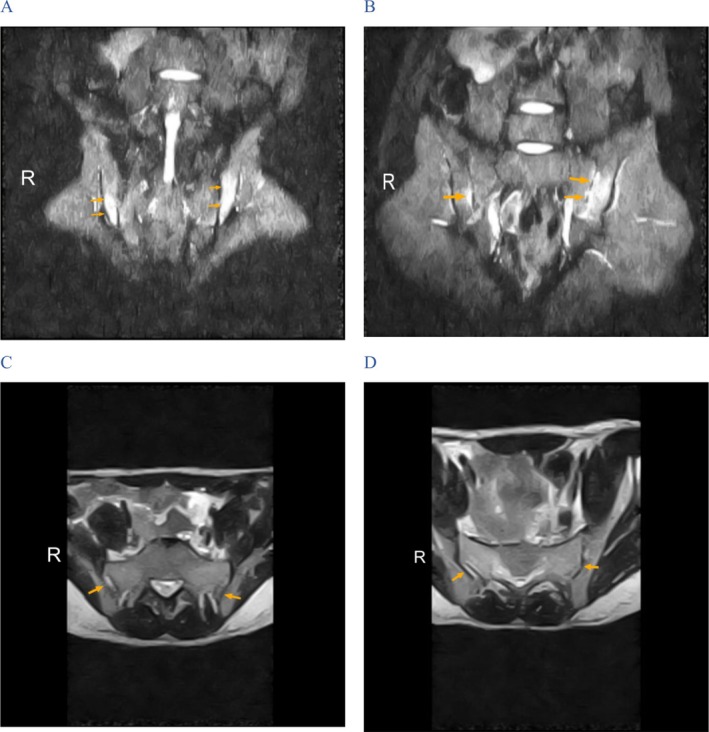
Magnetic Resonance Imaging (MRI) of the pelvis shows bilateral asymmetric subchondral bone marrow edema, more pronounced on the left, with erosive changes compatible with active sacroiliitis. (A) STIR coronal spot image showing asymmetric hyperintensities around sacroiliac (SI) joints, predominantly on the iliac side of the joint (yellow arrows), representing marrow edema. (B) STIR coronal spot image showing bone marrow edema (yellow arrows) around bilateral SI joints, more pronounced on the left side. (C) T2 axial spot image showing subtle hyperintensities around the SI joints bilaterally (yellow arrows), representing bone marrow edema. These changes are more pronounced on the left side and on the iliac side of the SI joints. (D) T2 axial spot image showing subtle bone marrow edema (yellow arrows), more pronounced on the left side. The rest of the sacrum appears normal.

### 2.4 Treatment

Considering the disease stage and risk of potential complications, the patient received a combination antibiotic therapy comprising oral doxycycline (100 mg twice daily), intravenous gentamicin (5 mg/kg/day), and oral co‐trimoxazole (160/800 mg twice daily) during his in‐hospital stay (day 1–5). Rifampicin, along with doxycycline, is the preferred combination for Brucellosis. However, rifampicin could not be used due to the lack of availability in the region. Alternatively, co‐trimoxazole—because of its high bone and synovium penetrance—was used as mentioned in the established literature [6, 9]. The patient responded well to the treatment, and thus the same antibiotic therapy was continued as an outpatient. Due to the significant risk of renal and ototoxicity with prolonged gentamicin use, the patient was advised to perform renal function tests every fourth day and report to the hospital in case of any abnormality in the tests, as well as hearing and balance problems.

### 2.5 Follow‐Up and Outcomes

In the subsequent visits, the patient was clinically evaluated, and relevant investigations, including blood counts, inflammatory markers, and renal function tests, were performed.

On a 10‐day follow‐up (day 15), the patient showed significant improvement—indicated by clinical evaluation, laboratory investigations (Table 1), and no reported medication‐related side effects, including vestibular symptoms, hearing changes, or reduced urine output. He was afebrile, denied any difficulty in walking, and reported improvement in pain. Considering the causative agent, the patient's prolonged history, and osteoarticular involvement, gentamicin was recommended for another 1 week, making the total duration 22 days (5 days in‐hospital + 17 days after discharge), while doxycycline and co‐trimoxazole were continued for another 11 weeks after follow‐up, making the total planned oral therapy duration 13 weeks.

On his second follow‐up (day 45), there were no significant findings on clinical evaluation, and his laboratory values had been normalized. The treatment plan was continued as planned.

A summary of key clinical events has been presented in Table 2.

**TABLE 2:** Chronological timeline of clinical events, investigations, and management.

Time frame	Event
At presentation (Day 1)	A 19‐year‐old male patient, with a history of unpasteurized milk intake, presented with bilateral buttock pain for 1 month, along with fever for the last 2 weeks.
Initial clinical evaluation	The patient had mild discomfort while walking. Direct sacroiliac compression, Patrick's, and Gaenslen's tests reproduced pain over both sacroiliac joints.
Laboratory and radiological investigations	Mild leukocytosis, elevated CRP, and ESR. Standard agglutination testing was positive for anti‐*Brucella* antibodies. MRI showed bilateral sacroiliitis with marrow edema and erosive changes.
Diagnosis	Brucellosis complicated by bilateral sacroiliitis.
Treatment and response (day 1–5)	Inpatient: started on combination antibiotic therapy, including oral doxycycline, oral co‐trimoxazole, and intravenous gentamicin. The patient showed optimal response with decreasing pain scores and no fever spike during hospitalization.
Afterwards (day 6 to 15)	Outpatient: discharged on the same regimen
1st follow‐up (day 15)	Improvement in pain and no medication‐related adverse effects.
Subsequent treatment (Day 15 onwards)	Gentamicin was recommended for another 7 days. Doxycycline and co‐trimoxazole were continued for another 11 weeks.
2nd follow‐up (day 45)	Normal findings on clinical evaluation and laboratory investigations.

## 3 Discussion

Brucellosis is endemic in rural and agricultural areas, particularly in the Mediterranean and South Asian regions. Unpasteurized dairy product consumption, as reported in our case, and direct contact with infected animals are the primary routes of transmission. Animal vaccination programs have greatly reduced the incidence of brucellosis; however, cases persist due to a lack of sufficient pasteurization and certain cultural beliefs that favor the consumption of raw dairy products [6].

Osteoarticular involvement is one of the most common focal complications of brucellosis, although its reported frequency varies depending on the study population, geographic region, and diagnostic approach [1]. Such involvement might be in the form of arthritis, spondylitis, and sacroilitis [6]. Many published case reports describe brucellosis presenting with septic arthritis and cutaneous manifestations [10]. The organism seeds the joint synovium via hematogenous spread and causes inflammation of the joint, articular surface erosion, and marrow edema. The sacroiliac joint involvement is typically unilateral, causing unilateral sacroiliitis, and the patient presents with unilateral hip or lower back pain. Bilateral involvement is less typical. Clinical presentation of bilateral cases, as seen in our patient, aligns more with tuberculous and autoimmune spondyloarthropathies like ankylosing spondylitis, posing diagnostic and therapeutic challenges for clinicians [1, 7, 11].

It is particularly concerning due to the usual high ratio of misdiagnosis in cases of brucellosis. A China‐based retrospective study analyzing 2060 patients with brucellosis reported that more than half (58%) of the cases had been misdiagnosed or suspected of other diseases due to a similar clinical presentation [12]. Although this finding highlights the misdiagnosis encountered in a particular cohort and such misdiagnosis rates are likely influenced by factors such as local healthcare infrastructure, access to diagnostic tools, clinician awareness, and referral practices, it still gives us a rough idea of the possible risk of misdiagnosis in uncomplicated brucella cases. It is very likely that these rates might increase when brucellosis presents unusually, such as in the form of bilateral sacroiliitis. Accordingly, brucella‐associated bilateral sacroiliitis should be considered a possible differential in patients presenting with bilateral buttock pain, particularly with prior exposure history or individuals from endemic regions, to prevent misdiagnosis. Such cases should be thoroughly assessed via proper blood tests, serologic findings, radiologic examinations, blood cultures, PCR studies, and image‐guided biopsy [1, 7, 11].

While testing for anti‐brucella antibodies remains the preferred initial investigation, false‐negative results can occur depending on the specific assay, diagnostic cutoff, and disease stage. In one diagnostic study, the standard agglutination test at a threshold of ≥ 1:160 yielded 90% sensitivity among confirmed brucellosis cases, indicating that 10% of cases were missed at this cutoff. Alternatively, PCR and culture studies are better confirmatory tests, yielding a definitive diagnosis. However, these are not always available in resource limited settings, as in our case. Similarly, an image‐guided biopsy of the joint can be performed, and samples can be Gram‐ and/or ZN‐stained, cultured, or sent for histopathology. It will help exclude tuberculous origin and establish a definitive diagnosis [9, 13, 14]. However, these were not feasible in our settings, and the patient's diagnosis was supported by exposure history, elevated inflammatory markers consistent with active inflammation, positive serology, and MRI findings.

MRI is the radiological investigation of choice for early detection of sacroiliac joint involvement. MRI findings—including articular surface erosions, bone marrow edema, and associated iliopsoas muscle edema—are, however, nonspecific and may resemble pyogenic as well as inflammatory etiology. But it is helpful to confirm and find the extent of joint involvement, as in our case [8, 13]. A notable proportion of brucellosis patients have subclinical sacroiliitis, and associated joint changes can be detected on MRI even in the absence of pain on presentation. Thus, MRI can be utilized to timely detect joint involvement and prevent morbidity associated with undiagnosed cases [15]. Additionally, the absence of paravertebral abscesses on MRI can help exclude tuberculous spondyloarthropathy, which typically presents with soft‐tissue abscesses [7].

Combination antibiotic therapy is used to treat adult brucellosis, aimed at enhancing intracellular bacterial clearance and reducing relapse. Commonly employed regimens include doxycycline plus rifampin. A multidrug antibiotic regimen is usually employed for at least 6 weeks, while extended treatment duration for focal or osteoarticular disease. Doxycycline, Rifampin, or both are very effective if initiated early, while co‐trimoxazole and fluoroquinolones are good alternatives. Aminoglycosides, especially gentamicin, are highly effective in preventing relapse of the disease [6]. In the present case, rifampin was not administered due to its unavailability. Our patient responded well to co‐trimoxazole in combination with doxycycline and gentamicin, aligning with the established literature. The effectiveness of the utilized therapy might be attributed to the high rates of bone and synovium penetrance of co‐trimoxazole and effective intracellular clearance of Brucella species by synergistic effects of doxycycline and gentamicin [9]. Early diagnosis and prompt therapy yield favorable outcomes, as demonstrated by our case at 10‐day follow‐up, and prevent chronic sequelae. Brucellar Sacroiliitis, if treated appropriately, shows symptom resolution over weeks and radiologic healing over months; however, if diagnosed late or treated inappropriately, it poses a risk of complications like chronic pain, joint destruction, and relapse. Relapse rate is nearly 15% in inadequately treated cases of brucellar sacroiliitis [16].

This case report has several limitations. Although the diagnosis was supported by an appropriate exposure history, clinical presentation, positive Brucella serology, elevated inflammatory markers, and MRI findings, confirmation through blood culture, PCR, and image‐guided biopsy remained lacking due to feasibility constraints in our settings. Therefore, tuberculosis or pyogenic sacroiliitis could not be excluded microbiologically. Also, long‐term relapse assessment was limited due to limited follow‐up data. Finally, this is a single case report and thus the findings cannot be generalized to all patients of brucellar sacroiliitis.

## 4 Conclusion

Clinicians should maintain a high index of suspicion for brucellar sacroiliitis in patients presenting with lower back or buttock pain from endemic regions with fever and a prior history of raw dairy intake or animal exposure. Usually unilateral, brucellar sacroiliitis can present bilaterally, mimicking tuberculous sacroiliitis or inflammatory spondyloarthropathy. Exposure history, targeted serologic testing, MRI findings, and culture or PCR when feasible are vital to the timely diagnosis and treatment of the patient. Early intervention comprising a combination antibiotic therapy yields a favorable response. Public health efforts to create awareness regarding livestock vaccination and proper milk pasteurization are necessary to reduce the burden of brucellosis.

## Author Contributions

**Zia Ullah:** conceptualization, methodology, data curation, investigation, writing – original draft, writing – review and editing. **Naeem Ullah:** conceptualization, writing – review and editing, supervision, project administration, data curation, validation. **Muhammad Kashif Habib:** methodology, validation, data curation, visualization, writing – review and editing. **Zain Ul Abedeen:** conceptualization, methodology, data curation, investigation, project administration, writing – review and editing, writing – original draft. **Abad Ur Rehman:** validation, visualization, writing – review and editing, data curation.

## Funding

The authors have nothing to report.

## Ethics Statement

Ethical approval was not required for this case report in accordance with institutional policies. It has been prepared in accordance with CARE guidelines for writing case reports, as per standard practice and journal requirements.

## Consent

Written informed consent was obtained from the patient for publication of this case report and accompanying images.

## Conflicts of Interest

The authors declare no conflicts of interest.

## Data Availability

The data that support the findings of this study are available on request from the corresponding author. The data are not publicly available due to privacy or ethical restrictions.
